# Tarsal fusion for pes equinovarus deformity improves gait capacity in chronic stroke patients

**DOI:** 10.1186/s12984-019-0572-2

**Published:** 2019-08-02

**Authors:** Jorik Nonnekes, Maartje Kamps, Jasper den Boer, Hanneke van Duijnhoven, Frits Lem, Jan Willem K. Louwerens, Noël Keijsers, Alexander C. H. Geurts

**Affiliations:** 1 Radboud University Medical Centre; Donders Institute for Brain, Cognition and Behaviour; Department of Rehabilitation, Nijmegen, The Netherlands; 2 Department of Rehabilitation, Sint Maartenskliniek, Nijmegen, The Netherlands; 3 Department of Orthopaedics, Sint Maartenskliniek, Nijmegen, The Netherlands; 4 Research Department, Sint Maartenskliniek, Nijmegen, The Netherlands

**Keywords:** Gait, Stroke, Tarsal fusion, Equinovarus, Rehabilitation

## Abstract

**Background:**

Gait impairments are common and disabling in chronic stroke patients. Pes equinovarus deformity is one of the primary motor deficits underlying reduced gait capacity after stroke. It predisposes to stance-phase instability and subsequent ankle sprain or falls. This instability is most pronounced when walking barefoot. Tarsal fusion is a recommended treatment option for varus deformity, but scientific evidence is sparse. We therefore evaluated whether a tarsal fusion improved barefoot walking capacity in chronic stroke patients with pes equinovarus deformity.

**Methods:**

Ten patients with a pes equinovarus deformity secondary to supratentorial stroke underwent surgical correction involving a tarsal fusion of one or more joints. Instrumented gait analysis was performed pre- and postoperatively using a repeated-measures design. Primary outcome measure was gait speed.

**Results:**

Walking speed significantly improved by 32% after surgery (0.38 m/s ± 0.20 to 0.50 m/s ± 0.17, *p* = 0.007). Significant improvement was also observed when looking at cadence (*p* = 0.028), stride length (*p* = 0.016), and paretic step length (*p* = 0.005). Step length on the nonparetic side did not change. Peak ankle moment increased significantly on the nonparetic side (*p* = 0.021), but not on the paretic side (*p* = 0.580). In addition, functional ambulation scores increased significantly (*p* = 0.008), as did satisfaction with gait performance (*p* = 0.017).

**Conclusions:**

Tarsal fusion for equinovarus deformity in chronic stroke patients improves gait capacity, and the degree of improvement is of clinical relevance. Our results suggest that the improved gait capacity may be related to better prepositioning and loading of the paretic foot, leading to larger paretic step length and nonparetic ankle kinetics.

## Introduction

Gait impairments are common and disabling in chronic stroke patients, as they result in reduced mobility, falls and fall-related injuries. [1, 2] Pes equinovarus is one of the primary motor deficits underlying reduced gait capacity in these patients. [3] It is the result of imbalance of ‘active’ muscle strength (weakness of dorsiflexors and evertors) as well as ‘passive’ muscle length and tone (contractures and spasticity of the plantarflexors and invertors) around the ankle and tarsal joints. Particularly the varus deformity of the hindfoot is disabling, as it predisposes to stance-phase instability and subsequent ankle sprain or falls. This instability is most pronounced when walking barefoot, e.g. when going to the bathroom at night, and imposes a heavy attentional load on patients to prevent ankle sprain or falling.

Clinical management of pes equinovarus is generally perceived as challenging. Although in the chronic phase after stroke balance control and balance correcting steps can be trained, [4] there is no evidence that exercise-based interventions can improve the gait pattern including pes equinovarus. The emphasis of management of pes equinovarus should therefore be on medical-technical interventions. [5] To improve management, we have recently introduced a step-wise approach to the treatment of pes equinovarus. [3] In this approach, we strongly suggest that, in the presence of a dynamic or fixed varus deformity of the hindfoot at initial contact or during the stance phase, surgical interventions need to be considered to restore a plantigrade foot position and improve stance-phase stability of the paretic leg. [3] Unfortunately, despite excellent practice-based experiences, the scientific level of evidence for these surgical interventions is very limited. [6] We therefore evaluated whether gait capacity in chronic stroke patients improves after a surgical correction of a pes equinovarus deformity. Specifically, we focused on surgical interventions that included a fusion of one or more tarsal joints. The tarsal fusion restores a neutral position at the hindfoot (resolves the varus component) and could be combined with lengthening of the calf muscles in the presence of an additional fixed pes equinus component. We focused on barefoot walking, as stance-phase instability due to varus deformity of the hindfoot is most pronounced then. Our primary outcome measure was self-selected gait speed, as this is often the most important treatment goal for patients. We hypothesized that self-selected gait speed would increase after tarsal fusion in chronic stroke patients suffering from equinovarus deformity.

## Methods

### Patients and intervention

Between December 2014 and April 2017, we included chronic (> 6 months post onset) patients after supratentorial stroke (either ischemic or hemorrhagic) who were evaluated at the mobility outpatient clinic of the Radboud university medical center and the Sint Maartenskliniek for gait problems due to stance-phase instability related to pes equinovarus, and who subsequently underwent a surgical intervention including a fusion of one or more tarsal joints. We excluded patients who previously underwent orthopedic surgery of the ankle or foot. Ten patients with a dynamic or fixed pes equinovarus foot deformity due to stroke were included (see Tables 1 and 2 for patient characteristics and type of surgical intervention, respectively). Tarsal fusion was often augmented by (partial) lengthening of the Achillles tendon and/or by correction of flexion deformities of the toes.

**Table 1:** Clinical characteristics of the participants

Sex	3 M, 7 F
Type of stroke	7 ischemic, 3 hemorrhagic
Age at the time of stroke (years)	41 (24–52)
Time post stroke (years)	7 (1–24)
Age at the time of surgery (years)	48 (30–62)
Time between pre-operative gait analysis and surgery (months)	9 (5–14)
Time between surgery and post-operative gait analysis (months)	7 (2–11)
Motricity Index - lower extremity	57 (39–80)
Brunnström stage - lower extremity	4 (3–5)
Quantitative vibration threshold - ankle / foot	7 (5–8)
Modified Ashworth Scale triceps surae	2 (0–3)
Berg Balance Scale	48 (5–56)

Data are mean (range). Motricity Index - lower extremity (%). Brunnström stage - lower extremity (max range 1–6). Quantitative vibration threshold – ankle/ foot (max range 0–8). Modified Ashworth Scale – triceps surae (max range 0–5). Berg Balance Scale (max range 0–56)

**Table 2:** Surgical interventions

Patient	Surgical interventions
1	Talonavicular arthrodesis, interphalangeal arthrodesis of hallux, tenotomy of toe flexors
2	Talonavicular and calcaneocuboid arthrodesis, gastrocnemicus slide
3	Talonavicular arthrodesis
4	Talonavicular arthrodesis, gastrocnemicus slide, tenotomy of toe flexors II-V
5	Talonavicular arthrodesis, gastrocnemius slide, tenotomy of toe flexor II
6	Talonavicular arthrodesis, gastrocnemicus slide
7	Talonavicular arthrodesis, lengthening of the extensor hallux longus muscle, interphalangeal arthrodesis I-III, tenotomy of toe flexors
8	Talonavicular arthrodesis
9	Talonavicular arthrodesis, gastrocnemicus slide, osteotomy metatarsal I, tenotomy of toe flexors
10	Talonavicular arthrodesis, interphalangeal arthrodesis of hallux

Postoperatively, patients were treated in a cast without weight bearing. After 4 weeks, patients were permitted to bear weight in a below-knee walking cast, which was followed by a removable walker after 8 weeks. Complications did not occur. All patients gave their written informed consent, and the study was performed in accordance with the local ethical guidelines.

### Assessments and design

An instrumented gait analysis was performed pre- and postoperatively in every patient using a repeated-measures design. During these instrumented gait analyses, all patients were assessed barefoot. Four consecutive walking trials at self-selected speed over a trajectory of 10 m, in which the subject actually stepped on the force plates, were collected (an average of more than four walking trials was not possible due to difficulties with walking barefoot pre-operatively).

The instrumented gait analysis included kinematic and kinetic assessment. Reflective markers were placed at anatomical landmarks according to the full-body Plug-in-Gait model. [7] Marker positions were recorded by an 8-camera 3D motion analysis system (Vicon Motion Systems, United Kingdom) at a sample rate of 100 Hz. Ground reaction forces under both feet were recorded at a sample rate of 1000 Hz by two force plates (AMTI Custom 6 axis composite force platform, USA). Kinetics and kinematics were calculated with Vicon Clinical Manager software. Kinematic data were calculated using markers positions sampled over the 10 m walking trajectory; kinetic data were based on one step per foot during each trial. In one patient, kinematic data were missing on the nonparetic side.

Outcome measures included the following spatiotemporal parameters (which were calculated using the heel marker positions in the kinematic data): walking speed, cadence, stride length as well as step length and single-support time of both the paretic and nonparetic leg. Step-length asymmetry was quantified by using a step-length ratio defined as the difference in step length between the paretic and nonparetic side, divided by the average step length of the paretic and nonparetic side (positive values indicate a larger paretic step compared to the nonparetic step). In addition, we calculated the following kinematic and kinetic outcomes: range of motion at the ankle joint during the gait cycle, internal peak ankle moment, and peak ankle power of the paretic and nonparetic leg. All outcome measures were averaged over the 4 consecutive trials. At the same time, we collected clinical and subjective scores. Ambulation capacity was evaluated by an independent research assistant using the Functional Ambulation Categories (FAC). [8] In addition, patients were asked to express satisfaction with their gait capacity on a scale (1 to 10) before and after surgery. Moreover, we asked patients whether their capacity to walk barefoot had improved after surgery (yes/no).

### Statistics

The spatiotemporal, kinematic and kinetic parameters were analyzed using paired t-tests. We used a Wilcoxon test for the FAC scores and subjective rating of gait satisfaction. The α-level was set at 0.05.

## Results

### Spatiotemporal gait parameters

**Fig. 1:**
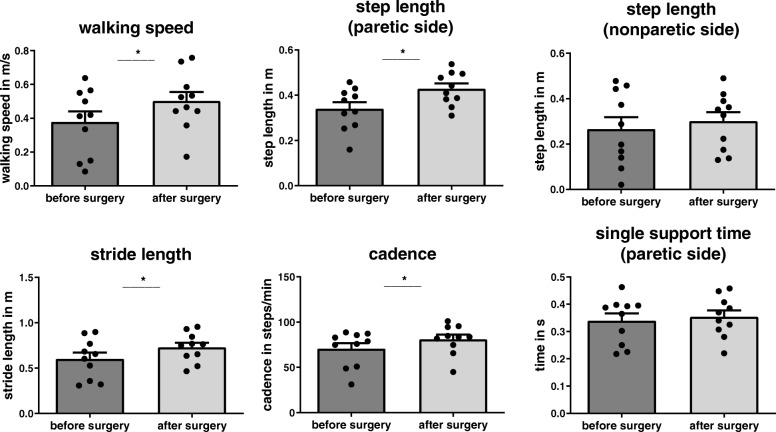
Values are averages with standard errors of the mean. * Significant differences (*p* < 0.05) before and after surgery

Self-selected walking speed improved significantly from 0.38 ± 0.20 preoperatively to 0.50 ± 0.17 m/s postoperatively (t [9] = − 3.492, *p* = 0.007). Improvement in gait speed was seen in 9 out of 10 patients. In one patient, even a fourfold increase in gait speed was found. Stride length also significantly improved from 0.60 ± 0.22 to 0.73 ± 0.16 m (t [9]= − 2965, *p* = 0.016) as did cadence from 69.8 ± 20.1 to 80.8 ± 15.4 steps/min (t [9]= − 2619, *p* = 0.028). Step length improved significantly on the paretic side (0.34 ± 0.09 to 0.43 ± 0.07 m; t [9] = − 3729, *p* = 0.005), but not on the nonparetic side (t [9] = − 0,942, *p* = 0.371; Fig. 1). Step-length ratio did not change significantly (0.34 ± 0.67 preoperatively, 0.41 ± 0.38 postoperatively; t [9] = − 0.330, *p* = 0.749). Single-support time did not significantly improve on either side (paretic: t [9] = − 0.433, *p* = 0.675; t [9] = − 0.581, nonparetic: *p* = 0.575). Figure 1 provides an overview of the changes in spatiotemporal gait parameters.

### Kinematic and kinetic parameters

**Fig. 2:**
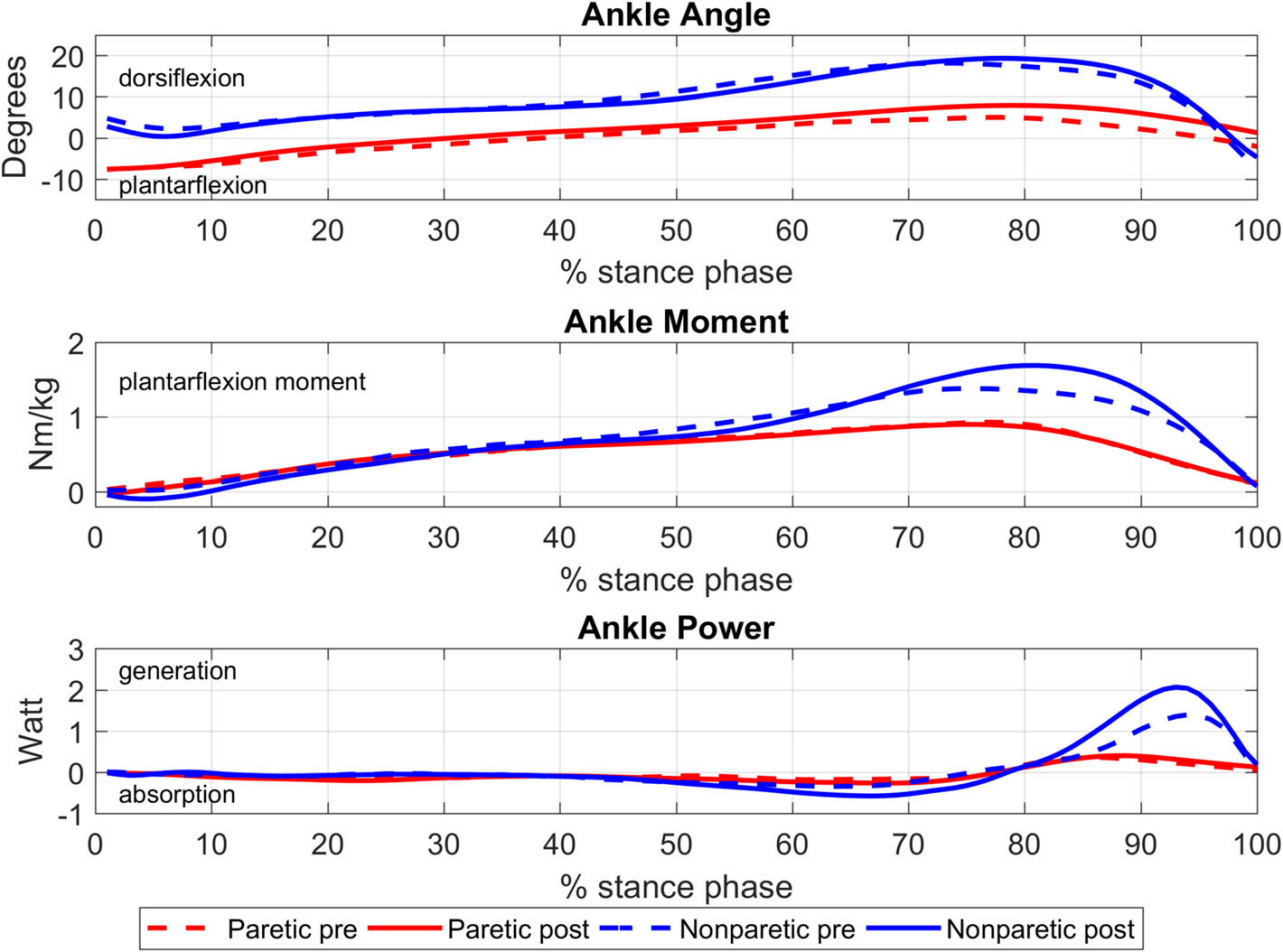
Mean ankle angle, ankle moment, and ankle power on the paretic and nonparetic side before and after surgery for all patients who were able to walk barefoot (*n* = 10)

Neither kinematics nor kinetics of the paretic side improved after surgery (range of motion ankle; t [9] = − 1.31, *p* = 0.220; peak ankle moment t [9] = 0.57, *p* = 0.580; peak ankle power t [9] = − 0.52, *p* = 0.620), but peak ankle moment on the nonparetic side increased significantly (from 1.14 ± 0.22 to 1.31 ± 0.26 Nm/kg, t [8] = − 2.87, *p* = 0.021), while peak ankle power showed a tendency towards significance (from 1.68 ± 0.75 to 2.35 ± 0.77 W, t [8] = − 2.14, *p* = 0.065) (Fig. 2).

### Clinical and subjective scores

Seven out of ten patients improved one FAC level (four patients from level 2 to 3, two patients from level 3 level 4, and one patient from level 4 to 5), while no one deteriorated (t [9] = − 4.583, *p* = 0.008). Preoperatively, patients scored their satisfaction with gait capacity 4.1 (range 2–7), which improved to 7.8 post-operatively (range 6–10; t [9] = − 4167, *p* = 0.017). Nine out of ten patients expressed a subjective improvement of gait capacity, whereas one patient scored slightly lower post-operatively (from 7 to 6). All patients reported an improved capacity to walk barefoot.

## Discussion

In this study, we evaluated whether surgical correction of pes equinovarus – involving a tarsal fusion of one or more joints – improves gait capacity in chronic patients after supratentorial stroke. After surgery, self-selected walking speed improved significantly, as did cadence and stride length. A significant improvement in step length was observed for the paretic leg, but not for the nonparetic leg. Peak ankle moment improved significantly on the nonparetic side, but not on the paretic side. Functional ambulation scores improved significantly, as did satisfaction with gait performance scored by the patients themselves.

This study specifically focused on tarsal fusion for pes equinovarus, whereas previous studies lumped surgical interventions for pes equinus and pes equinovarus. [6, 9, 10] However, the distinction between ‘pure’ pes equinus and pes equinovarus is important, as interventions for these conditions differ and concomitant varus deformity imposes a much greater threat on stance-phase stability and safety of gait than pes equinus alone. [3] Only one previous study specifically focused on a surgical intervention for pes equinovarus (SPLATT combined with Achilles tendon lengthening), [11] but this study did not quantify the effects on gait capacity. Hence, the present study is the first to objectively and specifically measure the effects of (augmented) tarsal fusion surgery in chronic stroke patients with a disabling pes equinovarus. When looking at gait speed, this intervention appears to be clinically relevant. Our population progressed on average from household ambulators (< 0.4 m/s) into limited community ambulators (0.4 to 0.8 m/s), [12] which transition has been associated with better daily life functioning and quality of life. [12] The presumed clinical relevance is further supported by better functional ambulation scores and patient-reported satisfaction scores. Perhaps most importantly, patients reported an improved capacity to walk barefoot. This gain renders them less dependent on an orthosis or orthopaedic footwear, which devices are commonly prescribed to patients with a disabling pes equinovarus. This reduced dependence on orthotic devoices may be of tremendous benefit for certain indoor (e.g. bathing and toileting) and outdoor (e.g. leisure) activities. However, this study was focused on gait outcomes observed in a laboratory; home and outdoor studies will be required to assess real world impact.

Why did gait capacity improve in our patients? Although several patients had interventions in addition to the tarsal fusion, such as Achilles tendon lengthening, we think the observed beneficial effects are mainly related to the tarsal fusion. Indeed, average range of motion at the ankle joint during the gait cycle – which might change after Achilles tendon lengthening – did not change significantly after the intervention. A fusion of one or more tarsal joints is thought to improve prepositioning during terminal swing (as there is no varus at the hindfoot anymore) and subsequent loading of the paretic foot. On the one hand, improved foot prepositioning and loading may allow a longer step length on the paretic side, as the risk of ankle inversion at initial contact is avoided. In this case, one could, in addition to an increase in paretic step length, expect an increase in ankle kinetics (‘push-off’) on the nonparetic side, as an increase in push-off is able to produce the kinetic energy to make a larger step. On the other hand, during stance phase the normal anatomical position of the hindfoot remains secured, and this improved stance-phase stability could lead to a longer single-support time on the paretic leg allowing a longer nonparetic step. Our results suggest that the first mechanism is more likely to occur, since paretic step length significantly improved and coincided with improved ankle kinetics on the nonparetic side, whereas single-support time on the paretic leg did not improve. Hence, the increase in walking speed was likely the result of improvement in non-paretic propulsion, which was possible because prepositioning of the paretic leg during terminal swing improved reducing the risk of ankle sprain at inititial contact. Why did single-support time on the paretic leg not improve? Probably, single-support time on the paretic side depends on many other factors than plantigrade foot positioning, such as knee and hip stability and the quality of postural control strategies exerted by the paretic leg.

This study has several limitations. The sample size was relatively small. Our results, therefore, need to be confirmed by future controlled studies with a larger sample size. As snapshot evaluations in a gait laboratory have inherent limitations, these studies should also monitor gait activity in daily life situations using wearables. Ideally, these future studies could further substantiate the positive effects of tarsal fusion by taking personal goals into account as well, in addition to measures of hindfoot positioning during terminal swing and plantar contact during the stance phase to evaluate outcomes from a biomechanical point of view.

## Conclusions

Tarsal fusion for equinovarus deformity in chronic stroke patients improves gait capacity. The degree of improvement is of clinical relevance. Tarsal fusion should therefore be considered to improve gait capacity in chronic stroke patients with equinovarus deformity.
